# Causes and effects of hospital nursing shortages to consider potential feedback effects: an umbrella review

**DOI:** 10.1186/s12960-025-01028-w

**Published:** 2025-11-06

**Authors:** David Jones, Sara Allin

**Affiliations:** 1 Institute for Pandemics, Munk School of Global Affairs and Public Policy, University of Toronto, Toronto, Canada; 2 Institute of Health Policy, Management and Evaluation, Dalla Lana School of Public Health, University of Toronto, Toronto, Canada

**Keywords:** Health policy, Human resources for health, Health workforce, Health services administration and management, Workforce shortage, Hospital, Nurse, Feedback, Public policy

## Abstract

**Background:**

In Canada and internationally, health systems have experienced rising healthcare staffing shortages in recent years. Specifically, this study seeks to analyse evidence on the causes and effects of hospital nursing shortages, to consider whether shortages may be self-reinforcing. It complements an existing linear healthcare workforce logic model (Sonderegger et al., 2021) by considering whether there may be evidence that implies the existence of feedback loops (a form of system dynamics).

**Methods:**

An umbrella review was undertaken to identify both causes and effects of hospital nursing shortages. A two-phase approach was undertaken: first, a review of all articles to identify a common list of factors, and second, a subsequent line-by-line review to ensure comprehensive coding.

**Results:**

The umbrella review identified several specific issues which were both causes and effects of nursing shortages, across a number of articles. This suggests that shortages could be self-reinforcing. For policymakers, the implication is that early intervention is likely to support the resilience and retention of hospital nurses. For researchers, this study highlights the risk of biased coefficients within econometric analysis and provides a testable cross-country hypothesis for the impacts of early intervention.

**Conclusions:**

Overall, this study contributes to existing academic literature and practical policymaking by identifying evidence that nursing shortages may be self-reinforcing. Through proactive intervention to restrain the growth of workforce shortages, policymakers can support the welfare of healthcare service users and nurses themselves.

**Supplementary Information:**

The online version contains supplementary material available at 10.1186/s12960-025-01028-w.

## Background

### Context

In Canada and internationally, health systems have experienced rising healthcare staffing shortages in recent years [1]. Staffing gaps grew during the period 2010–2020, but were exacerbated by the Covid-19 pandemic, both overall and for nurses specifically [2]. For example, in Canada, nurse vacancies roughly tripled during the period 2019–2022 [3].

Nursing shortages are currently a significant constraint to the delivery of safe, high-quality, accessible health care services, which has critical implications. First, shortages have a significant adverse impact on the welfare of patients [4]. For example, in Ontario, the most populous province in Canada, a shortfall in nurses has been cited as a key driver of temporary emergency room closures and has restricted healthcare access for the population [5]. Second, workforce shortages cause detriment to the wellbeing of existing staff. For example, in a 2022 study regarding the impact of the COVID-19 pandemic on Canadian critical care nurses, 100% of the 425 nurses surveyed had moderate-to-high burnout [6]. In light of these impacts, policymakers have responded to recent nursing shortages through a raft of measures, including financial incentives for nurse recruitment and retention [7, 8].

Helping policymakers to support the nursing workforce is relevant from several equity perspectives. Nurses were disproportionately vulnerable to infection and trauma during the Covid-19 pandemic, and faced a rise in abuse from patients frustrated at pressurised healthcare services [9]. Such vulnerabilities compound upon inequalities from the gender pay gap, given nursing is a female-dominated profession (92% in Ontario, 89% in England) [10–12]. Staff shortages often most acutely affect rural and remote areas (e.g. in emergency rooms), which typically have fewer staff and lower access to healthcare services [13].

Research is needed to explain the dynamics underpinning shortages and identify interventions and planning approaches that support workforce optimisation. Such research can support both proactive workforce planning and crisis management.

### Objectives of the study

There is already a considerable body of research available to support workforce public policy. For example, Park et al.’s (2019) systematic review of policies which seek to eliminate nursing workforce shortages finds evidence in support of policies which promote cross-sector co-operation, supported by government funding and systematic data collection [14]. Russel et al.’s (2021) systematic review of health workforce retention initiatives in rural and remote areas concludes that policymakers should seek to prioritise rural training pathways and educational interventions in order to increase retention of health professionals in rural areas [15]. This study seeks to provide additional insight by building on the existing literature. Specifically, this study analyses evidence on the causes and effects of hospital nursing shortages to consider whether shortages may be self-reinforcing.

This study has three main objectives. First, to complement existing workforce models by leveraging additional insights gained since the Covid-19 pandemic. Second, to support policymakers—in Canada and internationally—to achieve greater preparedness and resilience of healthcare services, which will improve patient welfare. Third, to enable governments to better understand the issues faced by nurses and therefore to better support their welfare as they deliver healthcare services.

## Methods

### Conceptual framework

The starting point for this study is Sonderegger et al.’s (2021) policymaker-facing model for human resources for health (HRH). This is an online tool, which is “a detailed, interactive logic model to map HRH [human resources for health] evidence and inform policy development and decision-making” [16] (p. 1).

Sonderegger et al.’s high-level HRH logic model (above) is a linear flow model—“from inputs to outcomes” (p. 13). It is a sequential model with five stages: contextual factors; health system factors; health workforce processes; health workforce outcomes; and health system outcomes. Within each of these stages, there are a number of detailed components. For example, the ‘Health workforce processes’ stage includes the following components: production (of staff); entry (into the workforce); maintenance and performance; enabling environment; and exit. The ‘linear flow’ nature of the model provides an understanding of the drivers and consequences for each stage.

The model intentionally excludes potential “feedback loops” (p.13)—a subset of system dynamics, whereby the outputs of a process subsequently impact upon the inputs—in order to avoid adding complexity within the model [17]. Whilst this simplification is helpful and justified in terms of providing policymakers with a practical planning tool, this study seeks to build on this framework to assess whether such feedback loops may or may not exist in practice. Were a feedback loop to exist, in practical terms this would mean that nursing shortages are self-reinforcing to some extent.

Whilst there is much literature that considers the causes and effects of workforce shortages—as discussed in the following sections—there has been only limited consideration of such feedback loops. One such example is a 2020 study (Farid et al.) which models system dynamics to show the effect of nurse workload on their health and on the quality of care [18]. The authors specify a causal loop, whereby staff shortages increase the workload of existing staff, cause fatigue and burnout, increase absenteeism, and subsequently increase (and exacerbate) the staff shortage.

To consider whether such feedback loops (a form of system dynamics) may exist in practice, the approach in this study was to undertake an umbrella review of academic articles on the causes and effects of nursing shortages and to review whether there is much overlap between the two. Specifically, if some of the effects (or resulting impacts) of nursing shortages are also causes, then that increases the likelihood that feedback effects exist and that—in practice—nursing shortages may be self-reinforcing.

This study complements the Sonderegger et al. model in two further ways. First, it is a more recent study, so it considers literature since the onset of the Covid-19 pandemic. This is relevant because the pandemic has exacerbated workforce shortages, as noted above, and therefore has increased the body of available evidence. For example, a recent study by Dill et al. (2022) found that existing short-staffing (largely due to pressures of working amidst the Covid-19 pandemic) was subsequently disincentivising entry by new staff, thereby reinforcing the shortage [19].

Second, it provides some indication of the materiality of the various causes and effects of shortages. The umbrella review in this study identifies the frequency with which different issues are identified. Whilst this is not a precise measure of materiality, it provides additional information for policymakers in understanding which are—from the academic literature—the most often-cited contributors to hospital nursing shortages. This therefore presents opportunities for further research.

Overall, this study adds value by leveraging Sonderegger et al.’s existing published tool and undertaking a deeper dive on specific areas that are likely to add material value for policymakers.

### Methods utilised for the umbrella review of relevant literature

This study specifically considers hospital nurses. Nurses are not a homogenous group, either within or across healthcare settings. For example, the issues affecting critical care nurses are very different from those facing nurses in long-term care or rehabilitation centres. To achieve internal validity and minimise variation, this study focuses on hospital nurses. This specific focus also has practical advantages for policymakers, because the shortage of nurses has been cited by many healthcare leaders as being the key driver for recent limitations in Canada’s hospital activities [20].

This study is authored from a Canadian perspective, and includes articles where the primary focus of analysis was Canada, or high-income countries (using the World Bank country classification), or a geographic area that included high-income countries [21]. This ensures a sufficient degree of comparability to Canada, and enables this study to be informative for Canadian policymakers, including government ministries and their agencies.

The study undertook an umbrella review, which is an analysis of existing systematic reviews. As systematic reviews typically already involve analysis of literature across multiple databases, the umbrella review was undertaken within the PubMed database, covering existing systematic reviews. This enabled the study to be undertaken efficiently, which supported the feasibility of research within an innovative field (i.e. feedback loops for nursing shortages). Whilst methodological guidance typically emphasises the importance of multi-database searches to reduce bias, Peters et al. (2020) state that searches should be “as comprehensive as possible within the constraints of time and resources” [22] (p.2123). The limitations of this search are discussed further below. To enable a wide coverage of potential issues, no time period constraint was applied. The following search term was used:

“( ( nurse ) OR ( nursing ) ) AND ( ( shortage ) OR ( shortfall ) ) AND ( hospital )”.

It is natural for organisations to have a certain level of vacancies, including due to natural attrition and organisational redesign to reflect evolving service requirements. Therefore, the terms ‘shortage’ and ‘shortfall’ were utilised—rather than ‘vacancies’—in order to more precisely target literature focused on large (or rising) vacancy levels, which were of greatest interest to this study.

The inclusion criteria for systematic reviews were as follows:

- Where the primary focus was hospital nursing shortages.
- Where the primary focus of analysis was Canada, or high-income countries, or a geographic area that included high-income countries—to ensure sufficient comparability to Canada. Other high-income countries were also included in the study—rather than solely Canadian studies—in order to generate a sufficiently large sample size.

**Fig. 1:**
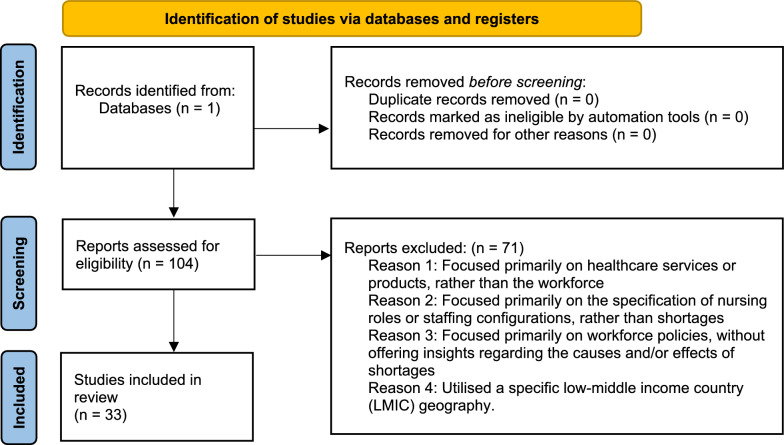
PRISMA diagram

The search generated 104 articles. Each of the abstracts of these 104 articles was reviewed for relevance to this study and 71 articles were excluded. The exclusion criteria are identified in the PRISMA diagram below (Fig. 1).

The remaining 33 articles were reviewed in detail to identify both causes and effects of hospital nursing shortages. This process was undertaken manually, without computer software, to facilitate intelligent review and interpretation of the literature. For example, when reading through a given article, if a particular issue was stated as a causal or contributing factor towards creating or exacerbating a nursing workforce shortage, that issue would be recorded as a cause.

Across the 33 articles selected for the umbrella review, a two-phase approach was used to identify causes and effects. Initially, a first review of all 33 articles was undertaken. During this review, an inductive, qualitative approach was taken to develop a comprehensive ‘master list’ of causes and effects across all articles [23]. This master list was developed inductively from this first review of the 33 articles to minimise the potential for pre-judgements or bias. This increased the accuracy and replicability of this study.

Subsequently, a second detailed review was undertaken—article-by-article—to identify which causes and/or effects from the ‘master list’ were identified in that article. The advantage of this two-phase approach was to ensure consistency across the articles in terms of classifying the causes and effects. The two-phase approach also achieved a high level of rigour by minimising the likelihood that a cause or effect was missed. By identifying the ‘master list’ within the first review, this increased confidence that causes and/or effects were identified comprehensively and precisely during the second review.

## Results

The results are recorded within data extraction tables (see supplementary materials). Table A identifies causes of nursing shortages by article, while Table B identifies effects of nursing shortages by article.

The articles varied in terms of their coverage and focus. Some studies were highly relevant to the focus of this study and identified multiple causes and/or effects of nursing shortages, whilst others provided more limited information. Of the 33 articles reviewed in this study: in four articles there were at least 20 causes or effects identified; in 10 articles there were 10–19 causes or effects identified; and in the remaining 19 articles there were fewer than 10 causes or effects identified.

Although most articles in the umbrella review focused on either causes or effects of nursing shortages, five articles specifically mentioned potential ‘feedback’ effects. For example, Toh et al. (2012) state that “findings revealed a positive bidirectional relationship between the nursing shortage and oncology registered nurses’ job dissatisfaction, stress and burnout” [24] (p.126). Bae’s (2023) analysis suggests that nursing turnover can be both a cause of nursing shortages and an effect [25].

Table 1 sets out the master list of causes and effects, and the number of articles (out of 33 articles in the umbrella review) in which each issue was identified as a cause or effect of hospital nursing shortages. The list of issues in the master list did broadly align with the high-level categories within Sonderegger et al.’s modelling, which is shown in Table C (supplementary materials). Tables A and B (supplementary materials) provide the article-by-article identification of causes and effects, which underpins the summary in Table 1.

**Table 1:** Number of articles (out of 33) in which specific causes and effects were identified Source: authors' analysis

Master list: causes and/or effects of hospital nursing shortages	Number of articles	
	Causes	Effects
Ageing population	9	0
Covid-19	10	0
General healthcare demand pressures	11	0
Political changes (i.e. Brexit)	1	0
Preparedness and planning	2	0
Training places, funding	6	0
Abuse by patients or other staff	5	2
Ageing workforce/early retirement	14	0
Culture/management/recognition/empowerment	14	3
High turnover/low retention	16	10
High turnover costs	0	8
Loss of organisational knowledge	0	1
Pay and other terms and conditions	10	0
Professional development and career opportunities	8	3
Quality/sufficiency/type of training	6	0
Support at work (incl. mentoring)	14	0
System/provider efficiency (incl. lost productivity)	0	11
Technicalities, e.g. credentialling	2	0
Understaffing/high workload	16	9
Working conditions/flexibility	16	1
Satisfaction/morale	16	10
Stress/burnout/mental health issues	18	15
Absenteeism	0	1
Occupational health issues/workplace injuries	2	0
Distress at health risks (e.g. Covid-19)	4	0
Waiting lists/ER closures	0	1
Universal healthcare coverage	0	2
Safety/quality of patient care	5	19
Patient outcomes, e.g. mortality	0	14
Patient dissatisfaction	0	1
Inequalities	0	5

Table 1 shows that the most frequently cited causes of nursing shortages were as follows: high turnover/low retention; understaffing/high workload; working conditions/flexibility; satisfaction/morale; and stress/burnout/mental health issues. Each of these issues was cited in at least 16 articles (out of the 33 articles in this study) (Fig. 1).

The most frequently cited effects of nursing shortages were as follows: high turnover/low retention; system/provider efficiency (including lost productivity); safety/quality of patient care; patient outcomes (e.g. mortality); satisfaction/morale; and stress/burnout/mental health issues. Each of these issues was cited in at least 10 articles (out of the 33 articles in this study).

**Fig. 2:**
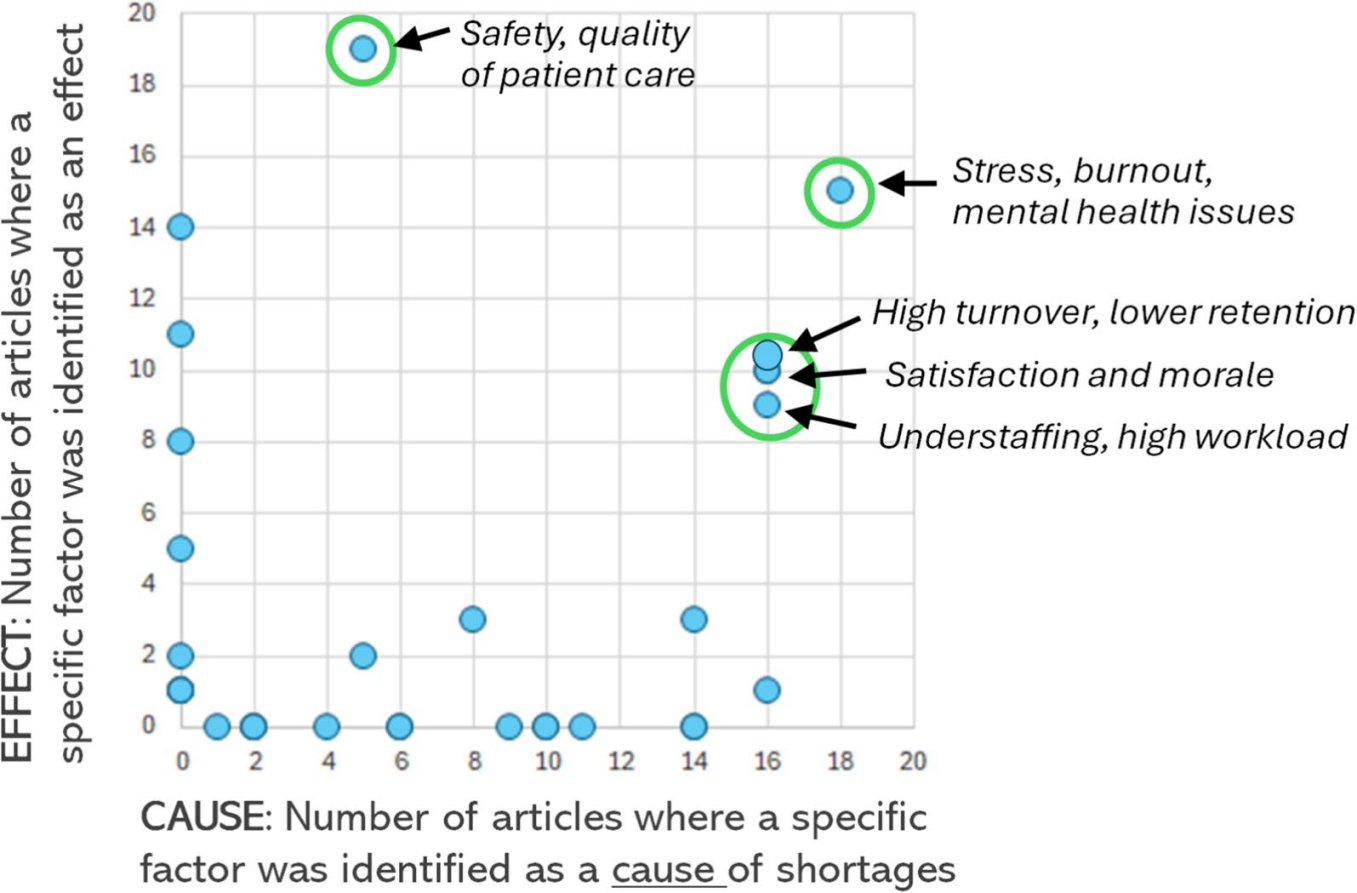
Number of articles in which each specific issue is cited as a cause (x-axis) or effect (y-axis) of hospital nursing shortages. Source: Authors’ analysis

Figure 2 (below) provides a scatterplot of Table 1 (above). Figure 2 shows—for each individual issue within the master list—the frequency (in terms of numbers of articles) in which each issue is identified as a cause and/or an effect of nursing shortages.

In Fig. 2, a data point somewhere along the x-axis represents an issue that is identified (only) as a cause of nursing shortages in one or more of the articles in the umbrella review. A data point somewhere along the y-axis represents an issue that is identified (only) as an effect of nursing shortages in one or more articles. Data points inside the chart have been cited as both a cause and an effect. Data points towards the top right-hand corner of the chart have—based on the umbrella review—a high frequency of being cited as both a cause and an effect.

For example, in Fig. 2, the data point closest to the top right-hand corner of the chart is *‘*Stress, burnout and mental health issues’. This was identified in 18 (out of 33) articles as a cause of shortages (x-axis), and in 15 (out of 33) articles as an effect of shortages (y-axis).

## Discussion

### Analysis of results

This umbrella review identifies many issues which are primarily either a cause of nursing shortages or an effect, but not both. However, a small group of issues are frequently identified as both causes and effects. This provides evidence that some effects of nursing shortages may also be causes of shortages, and vice versa. This suggests that feedback loops (or general equilibrium effects) may exist with respect to nursing availability and that—in practical terms—nursing shortages may be self-reinforcing to some extent.

As an example, the self-reinforcing nature of ‘Stress, burnout and mental health issues’ is a relatively intuitive result: stress and burnout are likely to increase staff attrition and lead to higher shortages; subsequently, this puts more pressure on the remaining staff (due to higher workload); and finally, this results in further stress and burnout for staff.

This evidence of potential feedback effects is beneficial in the context of the wider workforce modelling literature and planning. It provides methodological support for models that intentionally include feedback loops within their calculations for workforce supply and/or demand. For example, Ishikawa et al. (2013) have utilised system dynamics modelling to support forecasts for Japanese physicians and pharmacists [26, 27].

For researchers and policymakers, this study also emphasises the complexity of workforce shortages, which informs an understanding of the strengths and limitations of different modelling approaches. For example, whilst Sonderegger et al.’s linear logic model provides a clear framework for policymakers, it consciously excludes feedback effects. Strategic workforce modelling by policymakers often uses a linear approach—such as the NHS Long Term Workforce Plan (2023) [28]. Zhang et al.’s (2020) modelling analysis of physician shortages in the United States concludes that “increased efforts to understand shortage dynamics are warranted” [29] (p.1).

Moreover, evidence of feedback effects is useful in helping to anticipate the potential impact of shocks, such as the Covid-19 pandemic. Where feedback effects exist, the initial, direct impacts of a shock may be exacerbated by subsequent, indirect impacts.

### Policy and research implications

For policymakers, this evidence highlights the risk that shortages may be self-reinforcing, and potentially that shortages could lead to vicious spirals. This analysis suggests that policymakers can seek to systematically address the existence and/or development of system-level workforce issues through early policy intervention, which limits the potential for subsequent indirect effects, and by inhibiting the development of spirals.

For researchers, this study provides two useful implications. First, it highlights the need to account for indirect effects, to mitigate against biased coefficients within econometric modelling. Second, if positive feedback loops exist which exacerbate staffing shortages, then it would logically follow that those countries that have pursued proactive policy interventions (such as mental health supports for staff) may have experienced smaller workforce shortages than countries which were late to respond, ceteris paribus. This provides a testable hypothesis, which could be assessed via a cross-country econometric comparison.

For society more broadly, this study highlights the extreme pressures faced by nurses during the Covid-19 pandemic and therefore may support users of healthcare services to appreciate the work undertaken by this staffing group.

### Limitations

The umbrella review of articles in this study was undertaken manually, rather than with software. For example, the coding of causes and effects (for each article) required careful reading of each article’s text and subsequent judgement as to its meaning. The coding was undertaken by one team member, and the results were discussed with the research team. Manual coding ensured intelligent interpretation of the literature, although it required human judgement.

Similarly, the creation of the ‘master list’ of issues required some judgement in obtaining a list that was comprehensive, but not so granular as to be impractical. The two-stage approach to coding (outlined further above) was undertaken to increase objectivity and to comprehensively analyse the articles.

A research protocol was not included for this study, which limits transparency and reproducibility, so further research could benefit from introducing a relevant protocol.

This study undertook a review using the PubMed database and—after filtering against the study’s criteria—obtained 33 articles for review. There are pros and cons to including additional databases; Hartling et al. (2016) found that, for systematic reviews, the “majority of relevant studies can be found within a limited number of databases” [30] (p. 1). However, Goossen et al. (2020) highlight benefits from searching multiple databases when searching across health-related systematic reviews [31]. A follow-up study could consider widening the range of databases and reviewing a further set of academic articles, subject to meeting the relevant criteria.

The 33 articles analysed within this study include systematic reviews published both before Covid-19 and after Covid-19. Therefore, the results are at least partially generalisable.

The identification of optimal policy interventions is outside the scope of this review. The focus of this study is to support policymakers to better diagnose the key factors and dynamics around hospital nursing shortages.

Finally, this study offers a modest contribution to workforce planning and policymaking by targeting a specific area of analysis. Beyond this study, further research could consider alternative methodologies that can test for the presence and strength of feedback effects. If the body of evidence were to grow, subsequently consideration could be given to the relative merits of integrating feedback mechanisms into holistic workforce planning tools.

## Conclusion

Overall, this study contributes to existing academic literature by identifying evidence that hospital nursing shortages may be self-reinforcing. This highlights the importance of early intervention by policymakers in response to workforce shocks, such as the Covid-19 pandemic or otherwise. Through such proactive intervention, policymakers can support the welfare of healthcare service users and nurses themselves. This study provides a foundation to support further research regarding the presence and strength of feedback effects within hospital nursing shortages.

## References: Umbrella review

**Table:** 

Article	Reference
1	Tamata AT, Mohammadnezhad M. A systematic review study on the factors affecting shortage of nursing workforce in the hospitals Nurs Open. 2023 Mar;10(3):1247–1257. 10.1002/nop2.1434
2	Marufu TC, Collins A, Vargas L, Gillespie L, Almghairbi D. Factors influencing retention among hospital nurses: systematic review Br J Nurs. 2021 Mar 11;30(5):302–308. 10.12968/bjon.2021.30.5.302
3	Chan ZC, Tam WS, Lung MK, Wong WY, Chau CW. A systematic literature review of nurse shortage and the intention to leave J Nurs Manag. 2013 May;21(4):605–13. 10.1111/j.1365-2834.2012.01437.x
4	Wong CA, Cummings GG, Ducharme L. The relationship between nursing leadership and patient outcomes: a systematic review update J Nurs Manag. 2013 Jul;21(5):709–24. 10.1111/jonm.12116
5	Niskala J, Kanste O, Tomietto M, Miettunen J, Tuomikoski AM, Kyngäs H, Mikkonen K. Interventions to improve nurses’ job satisfaction: A systematic review and meta-analysis J Adv Nurs. 2020 Jul;76(7):1498–1508. 10.1111/jan.14342
6	de Vries N, Boone A, Godderis L, Bouman J, Szemik S, Matranga D, de Winter P. The Race to Retain Healthcare Workers: A Systematic Review on Factors that Impact Retention of Nurses and Physicians in Hospitals Inquiry. 2023 Jan-Dec;60:469,580,231,159,318. 10.1177/00469580231159318
7	Rodríguez-García MC, Márquez-Hernández VV, Belmonte-García T, Gutiérrez-Puertas L, Granados-Gámez G. Original Research: How Magnet Hospital Status Affects Nurses, Patients, and Organizations: A Systematic Review Am J Nurs. 2020 Jul;120(7):28–38. 10.1097/01.NAJ.0000681648.48249.16
8	Gualano MR, Sinigaglia T, Lo Moro G, Rousset S, Cremona A, Bert F, Siliquini R. The Burden of Burnout among Healthcare Professionals of Intensive Care Units and Emergency Departments during the COVID-19 Pandemic: A Systematic Review Int J Environ Res Public Health. 2021 Aug 2;18(15):8172. 10.3390/ijerph18158172
9	Yatsu H, Saeki A. Current trends in global nursing: A scoping review Nurs Open. 2022 May;9(3):1575–1588. 10.1002/nop2.938
10	Varghese A, George G, Kondaguli SV, Naser AY, Khakha DC, Chatterji R. Decline in the mental health of nurses across the globe during COVID-19: A systematic review and meta-analysis J Glob Health. 2021 Apr 10;11:05009. 10.7189/jogh.11.05009
11	Li H, Shi Y, Li Y, Xing Z, Wang S, Ying J, Zhang M, Sun J. Relationship between nurse psychological empowerment and job satisfaction: A systematic review and meta-analysis J Adv Nurs. 2018 Jun;74(6):1264–1277. 10.1111/jan.13549
12	Toh SG, Ang E, Devi MK. Systematic review on the relationship between the nursing shortage and job satisfaction, stress and burnout levels among nurses in oncology/haematology settings Int J Evid Based Healthc. 2012 Jun;10(2):126–41. 10.1111/j.1744-1609.2012.00271.x
13	Bae SH. Assessing the impacts of nurse staffing and work schedules on nurse turnover: A systematic review Int Nurs Rev. 2023 May 22. 10.1111/inr.12849
14	Lu H, Barriball KL, Zhang X, While AE. Job satisfaction among hospital nurses revisited: a systematic review Int J Nurs Stud. 2012 Aug;49(8):1017–38. 10.1016/j.ijnurstu.2011.11.009
15	Griffiths P, Saville C, Ball J, Dall’Ora C, Meredith P, Turner L, Jones J. Costs and cost-effectiveness of improved nurse staffing levels and skill mix in acute hospitals: A systematic review Int J Nurs Stud. 2023 Nov;147:104,601. 10.1016/j.ijnurstu.2023.104601
16	Bae SH. Assessing the relationships between nurse working conditions and patient outcomes: systematic literature review J Nurs Manag. 2011 Sep;19(6):700–13. 10.1111/j.1365-2834.2011.01291.x
17	Cowden T, Cummings G, Profetto-McGrath J. Leadership practices and staff nurses’ intent to stay: a systematic review J Nurs Manag. 2011 May;19(4):461–77. 10.1111/j.1365-2834.2011.01209.x
18	De Vries N, Lavreysen O, Boone A, Bouman J, Szemik S, Baranski K, Godderis L, De Winter P. Retaining Healthcare Workers: A Systematic Review of Strategies for Sustaining Power in the Workplace Healthcare (Basel). 2023 Jun 29;11(13):1887. 10.3390/healthcare11131887
19	Lartey S, Cummings G, Profetto-McGrath J. Interventions that promote retention of experienced registered nurses in health care settings: a systematic review J Nurs Manag. 2014 Nov;22(8):1027–41. 10.1111/jonm.12105
20	Liu S, Duan X, Han P, Shao H, Jiang J, Zeng L. Occupational benefit perception of acute and critical care nurses: A qualitative meta-synthesis Front Public Health. 2022 Sep 30;10:976,146. 10.3389/fpubh.2022.976146
21	Chan CW, Perry L. Lifestyle health promotion interventions for the nursing workforce: a systematic review J Clin Nurs. 2012 Aug;21(15–16):2247–61. 10.1111/j.1365-2702.2012.04213.x
22	Poon YR, Lin YP, Griffiths P, Yong KK, Seah B, Liaw SY. A global overview of healthcare workers’ turnover intention amid COVID-19 pandemic: a systematic review with future directions Hum Resour Health. 2022 Sep 24;20(1):70. 10.1186/s12960-022-00764-7
23	Ke YT, Kuo CC, Hung CH. The effects of nursing preceptorship on new nurses’ competence, professional socialization, job satisfaction and retention: A systematic review J Adv Nurs. 2017 Oct;73(10):2296–2305. 10.1111/jan.13317
24	Zulfiqar SH, Ryan N, Berkery E, Odonnell C, Purtil H, O’Malley B. Talent management of international nurses in healthcare settings: A systematic review PLoS One. 2023 Nov 6;18(11):e0293828. 10.1371/journal.pone.0293828
25	Dilig-Ruiz A, MacDonald I, Demery Varin M, Vandyk A, Graham ID, Squires JE. Job satisfaction among critical care nurses: A systematic review Int J Nurs Stud. 2018 Dec;88:123–134. 10.1016/j.ijnurstu.2018.08.014
26	Ong P, Cong X, Yeo Y, Shorey S. Experiences of nurses managing parenthood and career. A systematic review and meta-synthesis Int Nurs Rev. 2023 Sep 19. 10.1111/inr.12885
27	Gershon RR, Stone PW, Zeltser M, Faucett J, MacDavitt K, Chou SS. Organizational climate and nurse health outcomes in the United States: a systematic review Ind Health. 2007 Oct;45(5):622–36. 10.2486/indhealth.45.622
28	Gribben L, Semple CJ. Factors contributing to burnout and work-life balance in adult oncology nursing: An integrative review Eur J Oncol Nurs. 2021 Feb;50:101,887. 10.1016/j.ejon.2020.101887
29	Schaller A, Gernert M, Klas T, Lange M. Workplace health promotion interventions for nurses in Germany: a systematic review based on the RE-AIM framework BMC Nurs. 2022 Mar 21;21(1):65. 10.1186/s12912-022-00842-0
30	Allan JD, Aldebron J. A systematic assessment of strategies to address the nursing faculty shortage, U.S Nurs Outlook. 2008 Nov-Dec;56(6):286–97. 10.1016/j.outlook.2008.09.006
31	Xu G, Zeng X, Wu X. Global prevalence of turnover intention among intensive care nurses: A meta-analysis Nurs Crit Care. 2023 Mar;28(2):159–166. 10.1111/nicc.12679
32	Ding S, Deng S, Zhang Y, Wang Q, Liu Z, Huang J, Yang X. Experiences and needs of front-line nurses during the COVID-19 pandemic: A systematic review and qualitative meta-synthesis Front Public Health. 2022 Jul 22;10:805,631. 10.3389/fpubh.2022.805631
33	Wakefield E, Innes K, Dix S, Brand G. Belonging in high acuity settings: What is needed for newly graduated registered nurses to successfully transition? A qualitative systematic review Nurse Educ Today. 2023 Feb;121:105,686. 10.1016/j.nedt.2022.105686

## Supplementary Information

Supplemntary Material 1.

## Data Availability

All 33 articles included in the umbrella review are listed as references.
