# Supplementary material for: Causes and effects of hospital nursing shortages to consider potential feedback effects: an umbrella review
Source: Hum Resour Health. 2025 Nov 6;23:61. doi: 10.1186/s12960-025-01028-w (PMC12593840; doi:10.1186/s12960-025-01028-w)
Supplement: Supplementary file 1 — Supplemntary Material 1. [file 12960_2025_1028_MOESM1_ESM.docx]

**SUPPLEMENTARY MATERIALS**

**Table A: Data Extraction Table: Coding of causes of nursing shortages, by issue (rows) and by article (column)**

Source: Authors’ analysis

**Table B: Data Extraction Table: Coding of effects of nursing shortages, by issue (rows) and by article (column)**

Source: Authors’ analysis

**Table C: Mapping of this study’s ‘master list’ to categories within Sonderegger et al. (2021)**

| **Master list: Causes and/or effects of hospital nursing shortages** | **Sonderegger et al. categories** |
| --- | --- |
| Ageing population | Contextual factors |
| Covid-19 |  |
| General healthcare demand pressures |  |
| Political changes (i.e. Brexit) |  |
| Preparedness and Planning | Health system factors |
| Training places, funding |  |
| Abuse by patients or other staff | Health workforce processes |
| Ageing workforce / early retirement |  |
| Culture / management / recognition / empowerment |  |
| High turnover / low retention |  |
| High turnover costs |  |
| Loss of organisational knowledge |  |
| Pay and other terms and conditions |  |
| Professional development and career opportunities |  |
| Quality / sufficiency / type of training |  |
| Support at work (incl. mentoring) |  |
| System / provider efficiency (incl. lost productivity) |  |
| Technicalities, e.g. credentialling |  |
| Understaffing / high workload |  |
| Working conditions / flexibility |  |
| Satisfaction / morale | Health workforce outcomes |
| Stress / burnout / mental health issues |  |
| Absenteeism |  |
| Occupational health issues / workplace injuries |  |
| Distress at health risks (e.g. Covid-19) |  |
| Waiting lists / ER closures | Health system outcomes |
| Universal healthcare coverage |  |
| Safety / quality of patient care |  |
| Patient outcomes, e.g. mortality |  |
| Patient dissatisfaction |  |
| Inequalities |  |

Source: Authors’ analysis
